# Prospective Observational Study of Early Tracheostomy Role in Operated Severe Head Injury Patients at A Level 1 Trauma Center

**DOI:** 10.30476/BEAT.2021.86725.1198

**Published:** 2021-10

**Authors:** Rohit Bharti, Sindhu Sindhu, Ponraj K Sundaram, Ganesh Chauhan

**Affiliations:** 1 *Department of Neurosurgery, Rajendra Institute of Medical Sciences, Ranchi, India*; 2 *Northwick Park Hospital, London, UK*; 3 *Department of Neurosurgery, Goa Medical College, Goa, India*

**Keywords:** Head injury, Intensive care unit, Cerebral hypoxia, Tracheostomy, Glasgow coma scale

## Abstract

**Objective:**

To evaluate the impact of the early tracheostomy on operated patients with severe head injury.

**Methods:**

This prospective observational study was conducted at a level 1 trauma center and medical college over one-year period. The study included all surgically managed severe head injury patients without any other life-threatening major injuries. Patients who underwent tracheostomy within 7 days were classified as early tracheostomy.

**Results:**

The patient’s mean age of this cohort study was 43.4±14.5 years. Motor-vehicle accidents were being the most common cause of severe head injury. Operated patients were undergoing early tracheostomy on an average of 2.9 days. We were observed that the patients spent on a mechanical ventilation on an average 3.67±2.26 days. This was significantly lower than previous four published studies (*p*<0.05) which had a range of mean 9.8-15.7 days.

**Conclusion:**

We have shown that it is possible to decrease mechanical ventilation (MV) time, intensive care unit (ICU) stay and total hospital stay by doing early tracheostomy in operated severe head injury patients.

## Introduction

Traumatic brain injury (TBI) with a post-resuscitation glasgow coma score (GCS) of 3-8 is classified as a severe head injury (SHI). Both the primary brain injury sustained at the time of trauma and the secondary brain injury (SBI) triggered as a complex process after the primary injury contribute to the morbidity and mortality associated with TBI. Most of the mortality in these patients is due to cerebral hypoxia [1]. The outcome after SHI is often dismal and has a reported mortality of 37-51% [2, 3].

The severe head injury management essentially involves mitigating the SBI effects and nursing support for the unconscious patients. As hypoxia and hypotension are important causes of SBI, the management is of upmost importance in the conditions’ management. To prevent hypoxia, a proper airway and adequate ventilation are essential and the current paradigm for this is endotracheal intubation or tracheostomy along with mechanical ventilation. Usually, the patient is intubated and admitted in a critical care environment and supported with mechanical ventilation. Even after satisfactory weaning parameters achieving, the impaired level of consciousness and inability are strong reasons for delaying extubation and to protect their airway [4]. Then, trans-laryngeal intubation is changed to tracheostomy if the patient’s GCS continues to be below or equal to 8 and the patient remains ventilator dependent for more than 7 days [5]. There are no definite guidelines for the timing of tracheostomy [6].

There are several benefits associated with tracheostomy includes protection from laryngeal trauma, reduction of dead space, easy suctioning, better oral hygiene, and greater patient comfort [7, 8]. Early tracheostomy (ET) is performed based on clinical experiences as there is no fixed guidelines available [9-11]. An early tracheostomy would facilitate faster transfer of a patient from the intensive care unit once the respiratory parameters are normal. The purpose of this study was to evaluate the impact of the early tracheostomy on operated patients with SHI. We compared the duration of mechanical ventilation (MV), ICU and Hospital stay.

## Materials and Methods

This prospective observational study was conducted over one year at a tertiary care level 1 trauma center in India. The study included all surgically managed SHI patients without any other life-threatening major injuries such as blunt abdominal trauma, blunt chest trauma, multiple long bone fractures causing shock and cervical spine injury. Consent for the management was obtained from the immediate relative(s) of the patients. Institutional ethical committee (ECR/83/Inst/GOA/2013/RR-20) clearance for the study was taken.

All patients were managed according to the protocol described in protocol A in a neurosurgical ward. If the patient was directly transferred to the ICU from the emergency ward or after a surgical procedure, a tracheostomy was performed and the patient was transferred to the general neurosurgery ward; once the respiratory parameters were stable and the patient was breathing spontaneously. All the patients in the study underwent bedside tracheostomy during the management course. According to the tracheostomy timing, patients were classified as early group such as tracheostomy done in ≤7 days, or late (LT) such as >7 days. The outcome was assessed on the basis of a glasgow outcome scale (GOS) includes Grade I (death), Grade II (vegetative), Grade III (mostly dependent), Grade IV (minimally dependent), and Grade V (normal). Glasgow outcome scale (GOS) was assessed after one month at the time of hospital discharge. 

The means of various groups were compared using the mean of current study as reference, data on mean, standard deviation, therefore, number of patients were used as inputs to perform an unpaired t test using the online tool, graph-pad (www.graphpad.com). The *p* value < 0.05 was considered significant.

## Results

A total of 40 of operated SHI patients were included in the study. The age and sex distribution of the patients is shown in Table 1. The mean age of patients was 43.45±14.57 years (range 16 to 76 years). Patients aged between 31 and 40 years accounted for the majority (35%), followed by patients between 41 and 50 years old (22.5%). Elderly patients aged >60 years represented 12.5% of the total cases of TBI in this study. The men to women ratio was 7:1. The causes of the SHI were including 27vehicular accidents (67.5%); 11 for fall from height (27.5%); 1 assault (2.5%) and 1 unknown (2.5%). The mean time from trauma to arrival in our centre was 6.4 hours (median=4 hours, Interquartile Range (IQR):3-6). Twenty-three patients reached the hospital in less than 4 hours after trauma. Out of 40 patients included in the study, 15 (37.5%) had GCS ≤4. According to the admission GCS, the patients’ number is shown in Table 1.

**Table 1:** Data of Patients Included in Study

**Age Group (Years):**	**Male**	**Female**
11-20	3 (7.5%)	-
21-30	3 (7.5%)	-
31-40	14 (35%))	-
41-50	6 (15%)	3(7.5%)
51-60	6 (15%)	-
>60	3 (7.5%)	2 (5%)
Gcs^a^ at Admission:	GCS	Number of Patient
	GCS 3	6 (15%)
	GCS 4	9 (22.5%)
	GCS 5	9 (22.5%)
	GCS 6	2 (5%)
	GCS 7	4 (10%)
	GCS 8	10 (25%)
	Total	40
Glasgow Outcome Scale (GOS)	GOS^b^ At Discharge	Patients
	1 (Death )	18(45%)
	2 (Neuro Vegetative State)	1(2.5%)
	3 (Severe Disability)	20(50%)
	4 (Moderate Disability)	1(2.5%)
	5 (Good Recovery)	0 (0)
	GOS at 1 Month Follow Up	Patients
	1	0 (0)
	2	1(4.54%)
	3	4(18.18%)
	4	11(50%)
	5	5(22.72%)
	Lost To Follow Up	1(4.54%)
The Outcome of Treatment Depending on the Admission GCS		
GCS At Admission	Total Patients	Died
3	6 (15%)	5 (83.3%)
4	9 (22.5%)	5 (55.5%)^*^
>4	25 (62.5%)	8 (32%)^*^

^a ^GCS: Glasgow Coma Scale Score; ^b^GOS: Glasgow Outcome Score; *Number of patients with GCS=3 were used as reference. The number of patient with GCS=4 and GCS >4 were compared with GCS=3 and found to be not significant using Fischer exact test.

All the 40 patients underwent surgical procedure and the indications were acute 25 subdural hematoma patients (62.5%); 9 cerebral contusion patients (22.5%); 1 extradural hematomas patients (2.5%); 2 diffuse cerebral edema patients (5%) and 3 mixed findings patients (7.5%). 

Secure airway was immediately achieved on day 1 after trauma with EI (endotracheal intubation) or ET in 35 patients and in the remaining 5 on within 7 days after trauma. All the patients except two with GCS of 8 on admission underwent mechanical ventilation (MV) for at least 1 day. Tracheostomy was done in 40 patients; primary tracheostomy in 16 and conversion of EI to ET in 24. Tracheostomy was done on an average 2.9±1.75 days (median 3 (IQR:1-4.25)). All these patients underwent early tracheostomy before 7 days after admission. Mean duration of MV was 3.67±2.26 days (median 3 days (IQR:2-5)) which was significantly lower than other published study (Table 2). Tracheostomy could not be closed during the hospital stay in 1 patient. The average number of days to close tracheostomy in surviving patients was 12.80 days.

**Table 2:** Comparison of different studies with current study

**Study**	**N**	**Mean±SD**	***p*** **-value** ^a^
Duration on mechanical ventilation (in days)			
Current Study	40	3.675±2.26	Reference
Dunham *et al., *[34]	15	14.1±5.9	<0.0001
Wang *et al*., [38]	16	13.7±7.3	<0.0001
Teoh *et al*., [21]	15	9.8±5.9	<0.0001
Ahmad *et al*., [28]	27	15.7±6	<0.0001
Duration of ICU stay (in days)			
Current study	40	3.72±2.39	Reference
Wang *et al*., [38]	16	14.9+-8.9	<0.0001
Ahmad *et al*., [28]	27	19±7.7	<0.0001
Duration of hospital stay (in days)			
Current study	40	15.8±11.002	Reference
Wang *et al*., [38]	16	38±21.4	<0.0001

^a^p-value for difference of mean in comparison to mean of the current study using unpaired t test (t test function in www.graphpad.com)

Management Outcome

Amongst those who survived, 72.72 % had a good outcome with a GOS of 4 or 5 and were capable of independent self-care at one month after follow up (Table 1).

Duration of ICU Stay and Outcome 

In our study, the mean hospital stay for patients was 15.8±11.002 days (median 14 days (IQR:7.75-20.5)) and the mean ICU stay was 3.72±2.39 days (median 3.5 (IQR:2.5-5)) and they are being lower than most studies published in the literature (Table 2). Seventeen patients developed chest infection, out of whom 7 died during treatment course. Patients who developed chest infection during the course of treatment had an average hospital stay of 21.29 days. Total 29 patients were ventilated for more than 2 days, of whom 6 died due to chest infection.

GCS Score and Outcome

The outcome of treatment based on depending on the admission GCS is shown in Table 1. There was 83.3 % mortality in patients with GCS=3 and 55.5 % mortality in patients with GCS=4 on admission. In patients with GCS >4, the mortality was 32%.

## Discussion

TBI is a common cause of morbidity and mortality. In a recent study, severe TBI accounted for 14.3% of all admissions to ICU with a mortality rate of 54.0% and other studies have reported mortality of 37%-51% despite intensive management at tremendous cost [2, 3, 12]. The outcome depends on the extent of primary and the SBI. The primary injury occurs at the time of trauma and is seldom under the control of medical professionals. The SBI is a complex process initiated after the primary injury and is depending on several intracranial and extracranial processes of which can be modified with appropriate interventions and giving the opportunity for better clinical outcome with the right measures [13-17].

The intracranial SBI processes includes intracranial hematomas, cerebral edema, seizures, hydrocephalus, vascular injuries and infections. Extracranial processes causing secondary damage includes hypoxia, hypotension, hyperthermia, metabolic derangement and etc. [18]. The accepted management of Severe TBI involves admission in an ICU environment, endotracheal intubation initially with sedation and mechanical ventilation. Tracheostomy usually substitutes the endotracheal intubation subsequent usually after 7 days [5]. A sedated ventilated patient often requires ICU monitoring to assess neurological status and response to treatment.

Extubation timing or EI conversion to a tracheostomy is critical and dependent on neurological status, and the patient’s ability to maintain a proper airway. Most patients with severe TBI are at a high risk of morbidity and mortality if the ICU stay is prolonged [5, 19]. Patients who are extubated early on recovery after cessation of mechanical ventilation are unable to maintain a proper airway on account of altered sensorium whom they have a high frequency of reintubation on account of aspiration [20]. A tracheostomy is effective to maintain an airway and does not necessitate sedation, hence its timing would be very important to manage such patients.

In an intubated patient, the tracheostomy exact time has not been defined. Consensus conference on an artificial ventilation in 1989 recommended conversion to tracheostomy if mechanical ventilation (MV) was required for more than 21 days. Decision regarding tracheostomy timing varies across institutes. Presently, most physicians decide to tracheostomy time in patients with neurologic disease based on results derived from observations in patients mechanically ventilated for pulmonary causes. Most of the patients with severe TBI required intubation for airway protection more than supporting ventilation. Hence, ET provides an early alternative for airway protection and seems to decrease the need for prolonged MV [7].

This present study was designed to identify the impact of ET on the duration of MV, ICU stay, hospital stay, ventilator-associated pneumonia (VAP), and mortality, specifically for patients with isolated severe TBI. Most of the patient in our study group were between 31 to 40 year (n=14) age group followed by 41 to 50 years (n=11) with the mean age being 43.45±14.57 years. Men population was 7 times more involved in severe head injury than female population. 

We followed a policy of early tracheostomy where the endotracheal intubation was converted to a tracheostomy after an average of 2.9 days and always before 7 days that had elapsed after admission and intubation. Early tracheostomy helped initiate physiotherapy, decrease in pulmonary infection incidence and early mobilisation which helped to decrease morbidity and the mean hospital stay. The mean duration of mechanical ventilation was 3.675 days (SD=2.26days). The average time to close the tracheostomy in surviving patients was 12.80 days (SD=8.535 days). The mean hospital stay in our study group was 15.8±11.002 days. Both MV duration and Hospital stay were statistically significant when compared to different published studies.

Patients treated in our study group underwent ET. Among the 40 patients, 38 underwent MV for a variable period of time and 2 patients had ET but did not require MV. We noted ET helped weaning and transfer out of ICU. In the present study, mean ICU stay amongst surviving patients was 3.72±2.39 days which was less than most of the studies (*p*<0.0001) [21, 22]. By early weaning and transfer, we were able to prevent pulmonary complications and complications associated with endotracheal intubation. Endotracheal intubation is known to cause tracheal and vocal cord injury with poor oral hygiene and promotes aspiration. Accidental extubation are well known amongst this group. Patient’s transfer and care is difficult with tube in situ. In our study, we had two patients with stomal bleed that one was diagnosed later with dengue.

There is sufficient literature to support ET in ICU patients who fail to wean off the ventilator easily [8]. Tracheostomy not only provides a relatively stable and a well-tolerated airway but also provides access for good pulmonary toilet, makes oral feeding possible and permits earlier ambulation in turn to prevent orthostatic and Ventilated Associated Pneumonia (VAP) [23]. It also avoids any oropharangeal and/or laryngeal injury usually observed with EI [19, 24, 25]. Although several complications such as hemorrhage, tracheal stenosis and stomal infection have been reported but these are relatively infrequent [26]. 

Another study has shown that ET facilitates to decrease the MV duration. Lesnik *et al*., reviewed 101 adult patients who were admitted after blunt injuries. 32 had tracheostomy within the first 4 days and 69 underwent tracheostomy after 4 days. The authors found that the mean duration of ventilatory support was 6.0 days in ET group and was significantly less than the 20.6 days in the late tracheotomy group [23]. 

In the present study, the overall mortality was 45% but included 6 patients with an admission GCS of 3/15 who had fixed unreacting pupils. Of these 6 patients, 5 died and one was discharged with severe disability. There were 9 patients with GCS 4/15 among whom 5 died and 4 were discharged with a GOS of 3. These patients had presented early to casualty after trauma and had significant intracranial mass lesion or midline shift and hence were offered the benefit of surgery. In their surgery outcome study in patients with GCS of 3/ 15, Chamoun *et al*., [27] reported a mortality of 49.2 % and they argued for aggressive surgical intervention in these patients despite a poor account. There are other studies which have excluded patients with GCS 3 and 4 in calculating intervention outcome in Severe TBI. Excluding these 25 patients with GCS 3 and 4, the mortality in the remaining 25 patients was 32 % (8/25). 

The mean MV days for our patients was 3.67 days which is less than other reported studies [21, 22]. We were able to minimize ICU and ventilation times which had a great bearing on the outcome, minimising cost of treatment but at the same time not compromising on the treatment outcome. 

When ventilation was necessary for longer periods, respiratory complications were more frequent. Pneumonia was diagnosed in 43.5% patients on ventilator. In our group, 29 patients were ventilated for more than 48 hours and 17 patients developed pulmonary infection. Results were similar to the results achieved by Ahmed *et al*., [28, 29]. Siddiqui *et al*., [22] compared the outcome of ET against prolonged EI in 100 ICU patients with severe TBI who requires MV. They found that the mean time of MV was 10 days in ET group and 13 days for patients who were on EI. The VAP incidence was significantly higher in EI group, relative to ET group (63% vs. 45%, respectively). Due to the shorter ventilation and decreased incidence of VAP, patients in ET group were shifted out of ICU earlier as compared with EI group. The average ICU stay was 11 and 13 days in the ET and EI groups [22].

Bouderka *et al*., [30] conducted on severe TBI patients and patients were randomised to ET and prolonged EI group. The patients under ET group underwent tracheostomy on the 5^th^ or 6^th^ days. They did not find any differences in occurrence of nosocomial infection in the two group. They found the mean time of MV to be shorter in the ET group and that if nosocomial pneumonia developed, the number of MV days was more in the EI group. But there was no difference in the mortality in the two groups. Similarly, Rodriguez *et al*., [31] observed reduction in duration of MV, ICU, and hospital stay after ET in a homogeneous group of critically injured patients. Lesnik *et al*., [23] found that mean duration of MV in the ET and late tracheostomy group to be 6 days and 20.6 days, respectively.

ET also helps to minimize complication associated with prolonged EI. Nowak *et al*., [32] showed that tracheal complications were higher when EI was for more than 14 days as compared to EI for less than 14 days. In another study patient were divided into two groups: ET group (49 patients) (ET ≤7 days) and EI group (51 patients) (>7 days). They noticed increasing in VAP in EI group which was statistically insignificant and a significant decrease in MV time. The treatment cost was considerably less than the ET group patients [22]. In the retrospective study by Shibahashi *et al*., [33] the ET effectiveness was done within 72 hours which was assessed. ET group had 40 patients and late tracheostomy had 51 patients in groups. The duration of MV was significantly less in ET group, with no significant difference in adverse event rate. Some reports point out adverse consequences of ET. Dunham *et al*., [34] noted increase in in-hospital mortality in ET group when tracheostomy was done within 3-6 days after injury. A meta-analysis by McCredie *et al*., [35] found that ET done within 10 days of EI had reduced long term mortality. When ET is performed, it has been pointed out that these may be a case of unnecessary tracheostomy but ET helps to aggressive reduction of sedation and analgesic [35]. Baron *et al*., [36] concluded that patients with isolated TBI who underwent tracheostomy had a lower risk adjusted mortality rate as compared with intubated patients. Sheehan *et al*., [37] showed that similar findings of decreased ICU Stay and ventilator days when compared to LT.

On one month follow up, we observed that 16 patients (72 %) had good outcome with Glasgow Outcome Score (GOS) of 4.5. We found the substantial decreasing in the cost of ET treatment. But this did not increase the mortality, which was 32% in patients with GCS>4 and is comparable to reported mortality of 32-51 %. ET helped decreasing period of MV, ICU stay and hospital stay in the patients in our study.

In our prospective study, we have shown that it is possible to decrease MV time, ICU stay and total hospital stay by doing ET in operated severe head injury patients. Thus, it will benefit the patients by early discharge, early ambulation and better outcome.

In recent COVID 19 pandemic, the early tracheostomy role can be looked into and if similar results are reciprocated, therefore, it can be of utmost help to the strained health care system with insufficient ventilator bed. 
